# Diagnosis and treatment of chronic lymphocytic leukemia: 2025 recommendations of the Brazilian Group of Chronic Lymphocytic Leukemia of the Brazilian Association of Hematology and Hemotherapy (ABHH)

**DOI:** 10.1016/j.htct.2025.103822

**Published:** 2025-05-09

**Authors:** Carlos Sérgio Chiattone, Fernanda de Morais Marques, Valeria Buccheri, Mihoko Yamamoto, Sergio Costa Fortier, Maura Rosane Valerio Ikoma-Colturato, Nelson Hamerschlak, Vera Lucia de Piratininga Figueiredo, Talita Maira Bueno da Silveira, Abel Costa, Dani Laks, Rony Schaffel, Wolney Gois Barreto, Adriana Scheliga, Pedro Amoedo Fernandes, Samir Kanaan Nabhan, Rafael Dezen Gaiolla, Matheus Vescovi Gonçalves, Danielle Leão Cordeiro de Farias, Glaciano Ribeiro, Marcelo Pitombeira de Lacerda, Celso Arrais-Rodrigues, Brazilian Group of Chronic Lymphocytic Leukemia

**Affiliations:** a Brazilian Registry of CLL – Associação Brasileira de Hematologia e Hemoterapia, São Paulo, SP, Brazil; b FCM da Santa Casa de São Paulo, São Paulo, SP, Brazil; Hospital Samaritano Higienópolis, São Paulo, SP, Brazil; c ICESP - Faculdade de Medicina da USP, São Paulo, SP, Brazil; d Universidade Federal de Sao Paulo - UNIFESP/EPM, São Paulo, SP, Brazil; e Hospital Amaral Carvalho, Jaú, SP, Brazil; f Hospital Israelita Albert Einstein, São Paulo, SP, Brazil; g Hospital do Servidor Publico do Estado de Sao Paulo – IAMSPE, São Paulo, SP, Brazil; h AC Camargo Cancer Center, São Paulo, SP, Brazil; i Instituto D'Or de Pesquisa e Ensino, São Paulo, SP, Brazil; j Instituto de Hematologia, Porto Alegre, RS, Brazil; k Universidade Federal do Rio de Janeiro, Rio de Janeiro, RJ, Brazil; l Faculdade de Medicina do Unisalesiano, Araçatuba, SP, Brazil; m Grupo Oncoclínicas, Rio de Janeiro, RJ, Brazil; n Clínica AMO, Salvador, BA, Brazil; o Universidade Federal do Paraná, Curitiba, PR, Brazil; p Faculdade de Medicina de Botucatu (HC-FMB), Botucatu, SP, Brazil; q Grupo Fleury, São Paulo, SP, Brazil; r Beneficiência Portuguesa de São Paulo, São Paulo, SP, Brazil; s Universidade Federal de Minas Gerais, Belo Horizonte, MG, Brazil; t Universidade da Região de Joinville - UNIVILLE, Joinville, Santa Catarina, SC, Brazil; u Hospital Nove de Julho - DASA, São Paulo, SP, Brazil

**Keywords:** Chronic lymphocytic leukemia, Consensus, Treatment, Targeted therapies

## Abstract

Chronic lymphocytic leukemia, characterized by an accumulation of monoclonal B lymphocytes, is the most common adult leukemia. The disease predominantly affects older adults, with a significant proportion being asymptomatic at diagnosis. This manuscript provides a comprehensive review of chronic lymphocytic leukemia, including its epidemiology, clinical presentation, diagnostic criteria, and treatment strategies. Prognostic factors, particularly *IGHV* mutation status and chromosomal abnormalities, are discussed as critical determinants of disease behavior and treatment response. Recent advances in targeted therapies, such as Bruton's tyrosine kinase inhibitors (BTKi) and B-cell lymphoma 2 inhibitors (BCL-2i), have changed the treatment landscape by demonstrating superior efficacy to chemoimmunotherapy. However, disparities in access to care, particularly in low- and middle-income countries such as Brazil, highlight the need for equitable treatment approaches. The discussion of measurable residual disease (MRD) assessment for prognostication and treatment planning is also highlighted. This review highlights the need for continued research and integration of novel therapies to optimize patient outcomes in chronic lymphocytic leukemia.

## Introduction

Chronic lymphocytic leukemia (CLL) is the most common type of leukemia in adults, accounting for approximately 30 % of all leukemias in this population. The median age at diagnosis is 71 years, with >95 % of patients over the age of 50. While genetic and environmental factors may play a role in its development, the etiology of CLL is still unknown. The lower incidence of CLL in individuals of Eastern descent and its higher incidence in family members (5–10 %) when compared to other mature B-cell neoplasms reinforce possible genetic components in the development of CLL.

A first version of the Recommendations of the Brazilian Group of CLL was published in 2016.^1^ This updated second edition incorporates the latest therapeutic advancements, including novel targeted agents and combination regimens that have profoundly transformed the management landscape of CLL.

## Clinical presentation of CLL

The clinical presentation of CLL at diagnosis is highly variable. Approximately 60 % of patients are asymptomatic, with the disease often detected during routine blood work. When symptomatic, patients often report vague symptoms such as fatigue or weakness. Lymphadenopathy is observed in approximately 80 % of cases during the course of the disease, particularly in more advanced stages, often involving the cervical, axillary and inguinal lymph nodes. Splenomegaly is generally mild to moderate and occurs in about 50 % of cases, while hepatomegaly is less common. Although uncommon at diagnosis, B symptoms may be present as the disease progresses, defined as unintentional weight loss of 10 % or more in the past six months, fever above 38 °C for two or more weeks without other evidence of infection, and night sweats for more than one month without infection.

Anemia and thrombocytopenia may be seen in 15–30 % of patients, typically due to bone marrow (BM) infiltration. However, autoimmune cytopenias such as autoimmune hemolytic anemia and autoimmune thrombocytopenia may be present.^2^^,^^3^ Rarely, pure red cell aplasia and autoimmune granulocytopenia may be present. There is generally a good response to corticosteroids, but some patients require CLL-specific treatment for relapsed or refractory immune cytopenias. Other autoimmune manifestations are rarely seen in CLL patients, and may include myasthenia gravis, acquired von Willebrand disease and acquired angioedema The absolute lymphocyte count is highly variable both at diagnosis and over the course of the disease. Richter's transformation, formerly known as Richter's syndrome, is a condition that occurs when CLL transforms into an aggressive type of lymphoma, more commonly diffuse large B-cell lymphoma, or Hodgkin's lymphoma in a small subset of patients.^4^ Richter's transformation can be suspected by the appearance of B symptoms, rapid enlargement of the lymph node group, and marked elevation of lactate dehydrogenase.

### Central nervous system involvement in CLL

Central nervous system (CNS) involvement in CLL is rare but clinically significant, manifesting as confusion, cranial neuropathies, optic neuropathy, or cerebellar dysfunction. It can occur at any stage of the disease and may even be the first sign of progression requiring systemic treatment. In a recent analysis from the Brazilian Group of CLL, the most common presentations of CNS involvement were highlighted and relatively good outcomes were found, particularly with ibrutinib-based regimens.^5^^,^^6^ Given its potential impact, any neurological symptoms in CLL patients should prompt a thorough CNS evaluation to guide timely intervention.

Infection is the most common complication of CLL and the leading cause of death over the course of the disease.^7^ Both CLL itself and its treatment cause deficiencies in the cellular and humoral immune systems. Hypogammaglobulinemia is not uncommon and may worsen after CLL treatment. Bacterial infections are common even before treatment begins, with *Streptococcus pneumoniae* and *Haemophilus influenzae* being the most common pathogens. Response to vaccination varies and vaccination should be given early in the course of the disease for optimal results. Viral infections may also occur, with particular attention to herpes zoster reactivation. Hepatitis B and C virus reactivation may occur after treatment with immunosuppressive agents, including anti-CD20 antibodies. COVID-19 has also become an issue with a dismal clinical course in CLL patients, mostly during treatment and 6–12 months after anti-CD20 antibodies. Patients should be screened prior to initiation of therapy, and chronic hepatitis B virus carriers should be started on prophylactic antiviral therapy during CLL treatment, with entecavir being the drug of choice. The use of immunosuppressive agents such as corticosteroids, chemoimmunotherapy, and BTKi significantly increases the risk of opportunistic infections and invasive fungal diseases such as aspergillosis. Given the complexity of infection treatment and prevention in patients with CLL, it is advisable for the center to have an infectious disease specialist with expertise in oncohematology on staff.

Analysis of population-based data shows that patients with CLL have an increased risk of secondary cancers, with melanoma and squamous cell carcinoma of particular concern. They are also at higher risk for solid tumors, including colorectal, lung, kidney, thyroid and soft tissue sarcomas, than the general population. The occurrence of myeloid neoplasms was also elevated.

### Diagnosis

CLL is diagnosed by the presence of monoclonal B lymphocytes with a specific immunophenotype (CD5^+^/CD23^+^) in the peripheral blood (PB) at a count greater than 5 × 10⁹/L for more than 3 months.^8^ Below this threshold, it is considered monoclonal B-cell lymphocytosis (MBL) with a CLL phenotype, which can be further classified as high-count MBL (>0.5 × 10⁹/L) or low-count MBL (<0.5 × 10⁹/L). Despite its correlation with CLL, MBL is considered a distinct entity due to its extremely low progression rate and asymptomatic nature, with clinical management consisting only of periodic surveillance.^9^

Although the majority of high-count MBL cases have favorable prognostic features (*IGHV*-mutated and low-risk genomics), an estimated 1−2 % of individuals with high-count MBL will develop CLL requiring treatment.^10^

Small lymphocytic lymphoma (SLL) differs from CLL in the absence of leukemia, i.e., white blood cell count <5 × 10⁹/L, but requires lymphadenopathy and/or splenomegaly and lymph node biopsy for diagnosis. CLL and SLL represent different clinical manifestations of the same disease, distinguished only by the primary site of involvement: CLL predominantly affects the blood and BM, while SLL is characterized by nodal involvement with limited or no circulating disease. Despite these differences, both entities share identical biological, genetic, and prognostic features and should be managed identically.

In CLL, lymphocytes have a dense nucleus and lack visible nucleoli. The presence of 15 % prolymphocytes indicates prolymphocytic progression of CLL. Gumprecht shadows are common. Typical immunophenotypic markers include CD19^+^, CD5^+^, CD23^+^, CD200^+^, and CD43^+^, with weak expression of CD20, surface light chain (sIgκ^+^ or sIgλ^+^), and surface IgM, and weak or negative expression of CD79b, CD22^+^, and CD11c^+^, and absence of FMC7, CD10, and CD103. Historically, the Matutes scoring system based on five parameters (CD5^+^, CD23^+^, FMC7^-^, weak CD22/CD79b, weak K/L) was widely used for CLL diagnosis.^11^ Recently, standardized and internationally validated multicolor panels, including automated analysis, have gradually replaced its use.

Some cases of CLL exhibit atypical immunophenotypes, leading to diagnostic uncertainty. For example, high CD20 and FMC7 expressions have been associated with del(11q) and trisomy 12, while elevated IgM expression correlates with unmutated *IGHV* status and potential resistance to ibrutinib. However, despite this potential resistance mechanism, IgM expression is not currently used to guide treatment decisions. Nonetheless, monitoring IgM expression on CLL cells during ibrutinib treatment may serve as a biomarker for identifying the potential development of resistance.

### Differential diagnosis

The main differential diagnosis is mantle cell lymphoma (MCL): CD5^+^, but classically negative for CD23 and CD200 with strong expression of CD20 and immunoglobulins. The diagnosis of MCL is confirmed by FISH for t(11;14) or by immunohistochemistry for cyclin D1 or SOX11. Other B-cell lymphoproliferative disorders (BCLPD) may express CD5^+^, but usually at low intensity. In cases of uncertainty, diagnostic confirmation by cytogenetic, molecular or immunohistochemical methods is required, depending on the clinical context.

**Brazilian Group of CLL recommendations for diagnosis (mandatory)**

Morphological evaluation of PB smear.

PB immunophenotyping is essential for the diagnosis of CLL, including differential diagnosis with other BCLPDs, starting with a screening panel to determine the nature of the disease. The recommended diagnostic markers for CLL are CD19, CD20, CD5, CD23, CD200, CD79b, and kappa and lambda light chains. Other markers such as CD43, CD81, and ROR-1 and/or prognostic markers such as CD38, CD49b, or CD305 may be included. Depending on the flow cytometer available in each laboratory, 4, 6, 8 or more color panel combinations can be used, provided the protocol has been validated between laboratories. The Euroflow panel is an internationally validated 8-color approach that adheres to these recommendations and has been routinely used.

Cases with diagnostic uncertainty on immunophenotyping may benefit from additional diagnostic measures.

BM biopsy and/or aspirate immunophenotyping are NOT recommended for the routine diagnosis of CLL, but may be considered in cases of cytopenias to rule out myelodysplastic syndrome in clinical trials or in cases of diagnostic uncertainty.

Imaging modalities (ultrasound, computed tomography, magnetic resonance imaging, positron emission tomography scan) are generally NOT indicated in the diagnosis or initial assessment of CLL.

Alt-text: Unlabelled box

### Prognosis

First reported in the 1970s, the clinical staging systems (Rai and Binet, Table 1) are still widely used and are based on the assessment of nodal, splenic, and hepatic involvement, as well as cytopenias.^12^^,^^13^ Cytopenia in CLL predicts poor prognosis, though its impact depends on etiology. In a Mayo Clinic cohort, autoimmune-related cytopenia showed significantly better survival (9.1 versus 4.4 years, *p*-value <0.001) compared to BM failure.^14^

**Table 1:** Clinical stages and survival.

(A) Binet clinical stage (Binet et al.^13^)				
Stage	Risk	Characteristics (% of cases)	Median survival	
A	Low	< 3 areas of lymphadenopathy without anemia or thrombocytopenia (63 %)	15 years	
B	Intermediate	≥ 3 areas of lymphadenopathy^a^ without anemia or thrombocytopenia (30 %)	5 years	
C	High	Presence of anemia or thrombocytopenia (7 %)^b^	2 years	


(B) Rai clinical stage (Rai et al.^12^, Rai^15^)				
Stage	Risk	Characteristics	Median Survival (years)	Median Survival (years)
0	Low	Lymphocytosis	12.5	>13
I	Intermediate	Lymphocytosis + lymphadenopathy	8	7
II	Intermediate	Lymphocytosis + splenomegaly/hepatomegaly	6	
III	High	Lymphocytosis + anemia^c^	1.5	2
IV	High	Lymphocytosis + thrombocytopenia^c^	1.5	

a Bilateral cervical lymph nodes and Waldeyer's ring (one area), bilateral axillary (one area), bilateral inguinal (one area), palpable spleen and liver (one area each).

b Anemia: Hb <10 g/dL/Thrombocytopenia <100 × 10⁹/L.

c Anemia: Hb <11 g/dL/Thrombocytopenia <100 × 10⁹/L.

Immunophenotypic markers such as CD38, CD49d, CD305, CCR6, CXCR5, and ZAP-70 do not outweigh the impact of clinical staging and assessment of *IGHV* mutational status and abnormalities involving *TP53* despite their association with poor prognosis and chromosomal abnormalities.^16^^,^^17^ *IGHV* mutational status plays a critical role in prognosis: mutated *IGHV* is associated with a better prognosis and indolent course, while unmutated *IGHV* correlates with a more aggressive course.^18^ However, testing is not always accessible due to its high cost. Beyond its prognostic value, immunogenetic analysis has identified stereotyped B cell receptor immunoglobulin subsets, which define distinct clinical and biological CLL subgroups, refining risk stratification.^19^^,^^20^

Chromosomal aberrations, preferably detected through PB cytogenetics, are also useful for prognosis, especially when multiple abnormalities are present, as in complex karyotype.^21^ FISH detects aberrations in 80 % of cases, including del(13q14.1) (∼55 %), trisomy 12 (10–20 %), del(11q22–23) (10–25 %), and del(17p) (5–10 %). Complex karyotype and del(17p) are associated with unfavorable prognosis and may influence the choice of treatment. Monoallelic *TP53* mutations detected by polymerase chain reaction (PCR) or next generation sequencing indicate poor prognosis and resistance to therapy.^22^ *TP53* mutations and/or del(17p) are especially common in relapsed CLL and are associated with reduced overall survival.^23^, ^24^, ^25^ Other somatic gene mutations, including *ATM, NOTCH1, SF3B1*, and *BIRC3*, have been identified as prognostic markers, but only *TP53* is consistently associated with therapy resistance and early relapse.

Finally, the CLL-IPI score^26^^,^^27^ integrates genetic factors (*IGHV* mutation status, del(17p)/*TP53* mutation), clinical stage, age, and beta-2 microglobulin for prognostic assessment (Table 2). It is important to note that none of these factors indicates the need to start treatment in asymptomatic patients and that the prognosis associated with this staging and scoring system is related to an era of immunochemotherapy and has been modified with targeted therapies.

**Table 2:** Risk groups in CLL according to the International Prognostic Index (CLL-IPI) Criteria.

(A) Multivariate analysis of independent predictors for survival in CLL-IPI			
Variables	Risk factor	Relative risk	Score
Clinical stage	Binet B/C or Rai I-IV	1.6	1
Age	> 65 years	1.7	1
β2 microglobulin	> 3.5 mg/L	2	2
IgHV	Unmutated	2.6	2
Del17p and/or TP53 mutation	Deletion and/or mutation	4.2	4

(B) CLL-IPI risk groups according to overall survival			

Score (no. of unfavorable factors)		5-Year Survival (95 % CI)	
Low (0–1)		93.2 % (90.5–96.0)	
Intermediate (2–3)		79.3 % (75.5–83.2)	
High (4–6)		63.3 % (57.9–68.8)	
Very High (7–10)		23.3 % (12.5–34.1)

**Recommendations of the Brazilian Group of CLL for prognostic stratification**

In addition to clinical staging (Binet or Rai), the recommendations are to assess *IGHV* mutation status, and test for del(17p) by FISH, and TP53 mutation by PCR or next generation sequencing before initiating first-line treatment. If possible, detection of TP53 mutation and del(17p) deletion by FISH should be performed before starting each subsequent treatments because of the possibility of clonal selection after first-line treatment. There is no indication to repeat *IGHV* mutational status over the course of the disease.

Alt-text: Unlabelled box

### Measurable residual disease assessment

Assessment of MRD has gained importance following evidence that deeper remissions correlate with longer progression-free survival (PFS) and the ability of new regimens with immunotherapy and targeted therapies to induce high rates of undetectable MRD. MRD assessment after three and six cycles of disease eradication regimens and three months post-treatment appears to be an important predictor of CLL treatment outcome.^28^ As such, MRD assessment is increasingly being used in clinical trials in conjunction with traditional endpoints such as PFS and overall survival (OS).

MRD may also be an important predictor of outcome following hematopoietic stem cell transplantation. Both IgH-PCR and flow cytometry can be used to assess MRD.^29^^,^^30^ It is recommended that validated methods according to the protocols of the European Research Initiative on CLL (ERIC) and the European Study Group on MRD Detection (EuroMRD) be used.^31^ For both methods, the minimum sensitivity threshold of 1 × 10^–4^ (“MRD4”) is a key endpoint in several studies due to its reproducibility at this detection level and its prognostic correlation. Higher sensitivity levels (i.e., 1 × 10^–5^ to 1 × 10^–6^) can be achieved, but the clinical impact of lower thresholds is still being evaluated.

Flow cytometry is a widely used technique. The ERIC approach includes 6 or 8 markers (CD19/CD20/CD5/CD43/CD79b/CD81, with CD3 and CD22 for 8 colors). Sensitivity of 0.001 % (1 × 10^–5^) can be achieved with the detection of a higher number of events (at least 2 × 10^–6^ events). Other validated panel options include the 10-color panel from MD Anderson with automated analysis capability, the 12-color panel validated by Euroflow, and the 14-color panel validated by Memorial Sloan Kettering Cancer Center. These panels can be used according to the infrastructure available in each laboratory.

PCR for IGH regions can be used according to validated protocols (see EuroMRD). Real time quantitative-PCR (RQ-PCR) achieves MRD4 with good correlation to flow cytometry. However, challenges include the need for a specific laboratory infrastructure and the necessity (and difficulty) of obtaining the initial diagnostic sample to develop individualized primers. Newer, more sensitive techniques, such as next generation sequencing, are still under development, validation and international standardization.

Both PB and BM can be used to assess MRD. BM is more likely to be positive than PB. Therefore, if PB is negative, BM assessment may be used depending on the treatment goals. However, recent clinical studies are increasingly abandoning BM MRD evaluation due to its lack of clinical relevance, despite its slightly higher sensitivity compared to PB.

It should be noted that studies modifying subsequent therapy after MRD^+^ detection are still ongoing. Thus, despite its prognostic value, MRD assessment outside of clinical trials has no universal practical application as it does not guide treatment. The physician-patient relationship is critical to the assessment and interpretation of MRD outside of research contexts, as positive results may cause unnecessary distress. Furthermore, as demonstrated in the CAPTIVATE and FLAIR studies,^32^^,^^33^ MRD may be negative even in the context of partial remission or complete remission with incomplete hematologic recovery (CRi). MRD kinetics may vary depending on the therapeutic strategy used and should be evaluated and interpreted in the specific context of each treatment. Therefore, MRD^+^ is not synonymous with refractoriness, as patients may remain stable for years after treatment despite MRD^+^. In some cases, MRD^+^ may even become negative over time after treatment (e.g., the GLOW study).

**Brazilian group of CLL recommendations for MRD assessment**

Currently, the use of MRD assessment is NOT recommend in clinical practice. Routine MRD evaluation should be performed only in the context of research and clinical trials. In general, MRD assessment is conducted three months after completing therapeutic regimens aimed at eradicating leukemic clones (such as chemoimmunotherapy or venetoclax) and/or 12 months after hematopoietic stem cell transplantation.

The Brazilian Group of CLL recommends the use of standardized and validated protocols for MRD assessment, such as those established by ERIC and EuroMRD, considering the limit of detection (sensitivity) of the test.

Alt-text: Unlabelled box

### Treatment

The treatment of CLL/SLL has evolved significantly in recent years with the introduction of novel agents with targeted mechanisms of action.^34^ However, treatment indications remain those established by the International Workshop on CLL (iwCLL), mainly Binet C/Rai III/IV for patients with active and symptomatic disease. It is important to note that these recommendations have not changed with the introduction of targeted therapies (Table 3).

**Table 3:** Treatment indications according to IWCLL Criteria (2018) and the Brazilian Group of CLL treatment indications.

Criteria	iwCLL treatment criteria (2018)	Brazilian CLL group treatment indications
Bone marrow failure	Progressive bone marrow failure with anemia (Hb < 10 g/dL) and/or thrombocytopenia (platelets < 100,000/mm³)^a^	Progressive, symptomatic anemia and/or thrombocytopenia, persistent, excluding other causes
Splenomegaly	Massive (≥ 6 cm below the right costal margin), progressive, or symptomatic	Symptomatic splenomegaly
Lymphadenopathy	Lymph nodes ≥ 10 cm (longest diameter), progressive or symptomatic	Massive and symptomatic lymphadenopathy
Progressive lymphocytosis	Increase of ≥ 50 % in 2 months or lymphocyte doubling time (LDT) < 6 months^b^^,^^c^	–
Autoimmune complications	Anemia or thrombocytopenia with unsatisfactory response to corticosteroids	Autoimmune disease (anemia and/or thrombocytopenia) with inadequate response to corticosteroids or other treatments
Extranodal involvement	Symptomatic or functional extranodal involvement (skin, kidneys, lungs, CNS)	–
Constitutional symptoms	- Fever ≥ 38 °C for > 2 weeks without infection - Night sweats ≥ 1 month without infection - Weight loss ≥ 10 % in 6 months - Intense fatigue (ECOG ≥ 2)	- Significant unintended weight loss - Significant fatigue - Fever > 38.0 °C - Persistent night sweats (excluding other causes such as infection or neoplasms)

a Platelet values <100 × 10⁹/L may remain stable for long periods without requiring treatment.

b Exclude infection or corticosteroid use as a cause of lymphocytosis.

c Only consider LDT for lymphocytosis ≥ 30,000/mm³.

The isolated value of the absolute lymphocyte count, hypogammaglobulinemia, or monoclonal or oligoclonal paraproteinemia, should be interpreted in the context of a comprehensive clinical evaluation, rather than used as the sole indication to start treatment.

CLL and SLL should always be treated with the same therapeutic approach. SLL should not be treated as an indolent lymphoma because its natural history, treatment indications, and responses to targeted therapies are identical to those of CLL. Consistent application of CLL treatment paradigms to SLL will ensure optimal patient outcomes and prevent undertreatment due to misclassification of disease behavior.

The Brazilian Group of CLL performed an analysis of 2511 patients from 41 centers. Of these, 1404 patients (56 %) met the iwCLL indication criteria (liberal criteria), while only 788 patients (31 %) met the more restrictive Brazilian Group of CLL criteria. These criteria establish different cut-offs for cytopenias (hemoglobin <9.5 g/dL and/or platelets <50,000/mm³) and do not consider progressive lymphocytosis or disease-related symptoms for treatment initiation in the absence of cytopenias or symptomatic masses. Patients with liberal criteria had a better OS than those with restrictive criteria (85 % versus 68 %), suggesting that restrictive criteria are more predictive of prognosis than liberal criteria. Furthermore, among patients with liberal criteria, OS was significantly worse in treated patients (83 %) compared to untreated patients (97 %; *p*-value <0.0001), suggesting a possible detrimental effect of treatment in patients with borderline indications. The goal of this analysis was to suggest that treatment indication should only consider criteria that truly affect clinical outcomes and patient quality of life, thereby avoiding unnecessary treatments, costs, treatment-related toxicity, and potential interference with disease biology by selecting for more resistant clones (Table 3).

### Asymptomatic disease

To date, there is no evidence to support treatment of CLL at the time of diagnosis in the absence of symptoms. For patients with asymptomatic disease (Rai 0, Binet A) or asymptomatic intermediate-risk disease (Rai I-II, Binet B), watchful waiting with clinical assessments and blood counts every three months, especially during the first year, is recommended. Those with stable disease may be followed at longer intervals, from six to even 12 months (Table 4).

**Table 4:** Treatment recommendations from the Brazilian CLL Group.

### Symptomatic disease

Proper assessment of symptomatic disease is critical to select the most appropriate treatment for each patient. In addition to disease stage and cytogenetic risk, the patient's physical condition and comorbidities must be considered. A useful tool in this context is the Cumulative Illness Rating Scale (CIRS), which allows patients to be ranked in terms of treatment suitability according to known comorbidities.^35^ In clinical trials, patients with a CIRS score ≤6 and normal glomerular filtration rate (creatinine clearance >70 mL/min) are generally considered fit for more intensive treatments. It is important to note that age should not be used as a stand-alone marker of eligibility for CLL treatment, especially in the context of targeted therapies.

### Chemotherapy/Chemo-immunotherapy

Monotherapy with alkylating agents such as chlorambucil has been a common choice for many decades and may still be an option, especially for very elderly patients or those in poor health and unsuitable for more aggressive treatments. Chlorambucil offers notable advantages such as lower cost, low toxicity and ease of oral administration. However, its main disadvantages include very low complete remission (CR) rates and the risk of long-term side effects such as myelodysplasia. Nowadays, when available, chlorambucil monotherapy is avoided in favor of its combination with anti-CD20 monoclonal antibodies, which leads to improved response rates and PFS probabilities.

Fludarabine, a purine analogue, has been extensively studied in CLL. In various studies, response rates to fludarabine monotherapy range from 63 to 73 %, with 7–40 % of patients achieving CR. Combinations of fludarabine with cyclophosphamide (FC) have demonstrated better overall response rates (74–94 %) and CR rates (23–38 %) compared to other regimens in the pre-rituximab era, which marked the beginning of combination therapy.^36^

The introduction of chemoimmunotherapy further improved outcomes in the frontline setting. Studies such as CLL8^37^ demonstrated the superiority of the fludarabine, cyclophosphamide and rituximab (FCR) regimen over FC, providing higher response rates and prolonged PFS without increasing toxicity or the risk of infection. Long-term follow-up of the FCR arm showed prolonged OS, and in *IGHV*-mutated patients without del(17p), a survival plateau was observed, suggesting the potential for cure. This finding has been corroborated by other studies in FCR-treated patients.^38^^,^^39^ The FCR combination became the treatment of choice for patients eligible for intensive therapy. However, it is important to note that FCR is associated with an increased risk of myelodysplastic syndrome and acute myeloid leukemia compared to targeted therapies. Given the high prevalence of CLL in elderly patients, an FCR-Lite regimen was developed to reduce toxicity while maintaining efficacy.^40^

The CLL11 trial also showed promising results with the combination of obinutuzumab and chlorambucil, with a response rate of 78.4 % and a CR rate of 20.7 % in patients ineligible for fludarabine-based treatment.^41^ Obinutuzumab achieved better response rates than chlorambucil monotherapy and the rituximab/chlorambucil combination. It is important to note that obinutuzumab-related infusion reactions occur in approximately 65 % of subjects during the first cycle, with 21 % of these reactions being Grade 3 or 4, leading to discontinuation in 7 % of patients.

In a clinical trial, bendamustine, an alkylating agent with purine analog properties, was compared to chlorambucil and achieved better response rates (68 %) with a CR of 31 % and PFS of 21.6 months.^42^ The CLL10 trial showed that rituximab with a bendamustine dose of 90 mg/m^2^ in the first-line setting resulted in response rates similar to FCR at 97 %, but with fewer CRs (31 %).

Currently, FCR is an appropriate option for patients ≤65 years of age with creatinine clearance >70 mL/min, mutated *IGHV*, and without *TP53* alterations or complex karyotypes, when targeted therapies with or without anti-CD20 antibodies are not available, mostly in limited access scenarios. This fixed-duration therapy can produce durable remissions, some lasting more than 10 years, justifying its continued use as first-line therapy. Patients with mutant *IGHV* aged >65 years or ≤65 years with comorbidities (CIRS >6 and <12) can receive the bendamustine/rituximab regimen (BR) or chlorambucil with an anti-CD20 agent (obinutuzumab is the most active).

### Targeted therapies

Over the past decade, therapies targeting the B-cell receptor (BCR) or the anti-apoptotic protein B-cell lymphoma 2 (BCL-2) have profoundly transformed the treatment of CLL. These therapies include both continuous and fixed-duration regimens, all of which have demonstrated superiority over chemoimmunotherapy. This shift follows the FDA approvals of the covalent BTKi ibrutinib in 2014 and the BCL-2 inhibitor (BCL-2i) venetoclax in 2016.

Two second-generation covalent BTKis, acalabrutinib and zanubrutinib, have shown improved safety profiles compared to ibrutinib, with potentially lower toxicity, and were approved by the FDA in 2019 and 2023, respectively. With the availability of these agents, it has become clear that, in addition to clinical and molecular characteristics, other factors, such as specific comorbidities, concomitant medications, and therapy-related risks, should be considered when selecting the optimal first-line treatment for each patient.

For subsequent lines of treatment, it is crucial to assess the response or lack of response to prior therapy, duration of response, the development of resistance to a specific agent, or the occurrence of toxicity that prevents continuation, as well as the presence of *TP53* mutations or del(17p).

### *Covalent* Bruton's tyrosine kinase inhibitors

Through irreversibly binding to the cysteine residue (C481) in Bruton's tyrosine kinase (BTK) domain and inhibiting its enzymatic activity, covalent BTKi interferes with B-cell receptor signaling, affecting adhesion, migration, proliferation and cell survival, resulting in redistribution of CLL cells from secondary lymphoid organs to the PB, reducing lymphadenopathy and splenomegaly, with an expected transient increase in PB lymphocytes over the first weeks or months of treatment. Over time, lymphocytosis decreases due to deprivation of survival signals from lymphoid tissues and a direct pro-apoptotic effect. Despite favorable long-term outcomes, monotherapy with covalent BTKi does not induce deep molecular responses, with low rates of undetectable MRD. Ibrutinib was the first in this class to enter clinical trials and currently has the most extensive data of any available BTKi, particularly in high-risk patients. The pivotal phase 1b/2 study PCYC-1102/1103 in heavily pretreated and treatment-naive patients aged ≥65 years compared two doses of ibrutinib (420 mg versus 840 mg) with identical overall response rates of 71 % in both groups. This established 420 mg/day as the standard dose for CLL/SLL. In patients with del(17p), the response rate was similar at 68 %, highlighting the efficacy of ibrutinib in this poor prognostic group. These initial results have been confirmed in three additional studies.

The RESONATE study (PCYC-1112) in relapsed/refractory CLL/SLL [86 % high-risk alterations (del17p/*TP53* mutation), del(11q) and/or unmutated *IGHV*] showed significant improvements in PFS and OS with ibrutinib compared to anti-CD20 ofatumumab. With a median follow-up of 6 years, the median PFS remained significantly longer in the ibrutinib arm with continued OS benefit.^43^ The RESONATE-2 study (PCYC-1115/1116) in treatment-naive patients ≥65 years of age without del(17p) showed an OS benefit with a PFS rate of 70 % with ibrutinib versus 12 % with chlorambucil.^44^ Recent data show good tolerability of this agent, with 42 % of patients on continuous ibrutinib after 7 years of follow-up.^45^ Finally, the RESONATE-17 trial^46^ confirmed the efficacy of ibrutinib in previously treated patients with a median age of 64 years and del(17p). The 24-month PFS rate was 63 %, and 75 % of patients were alive at 2 years. The most common reasons for discontinuation were disease progression in 24 % of patients and adverse events with unacceptable toxicity (mainly arrythmias and infections) or death in 17 % of patients.

The multicenter Phase 3 ILLUMINATE study^47^ in previously untreated CLL/SLL patients aged >65 years or ≤65 years with comorbidities randomized patients to continuous oral ibrutinib plus obinutuzumab (IO) or chlorambucil plus obinutuzumab (CBL+O). At a median follow-up of 31.3 months, the median PFS was not achieved in the IO arm and was 19.0 months in the CBL+O arm, with 30-month PFS estimates of 79 % with IO and 31 % with CBL+O. The most common Grade 3 or 4 adverse events in both arms were neutropenia and thrombocytopenia. Serious adverse events occurred in 58 % of patients treated with IO and 35 % of patients treated with CBL+O.

The ALLIANCE 202 trial compared ibrutinib ± rituximab with BR in elderly patients with previously untreated CLL.^48^ PFS at 2 years was 74 % with BR and 87 % with ibrutinib monotherapy. No significant difference in PFS was observed between the ibrutinib + *R* and ibrutinib monotherapy groups. The PFS benefit of ibrutinib over BR was seen in all cytogenetic subgroups, with del(17p) being the most prominent. PFS differences were maintained at 4 years of follow up.

Two studies compared ibrutinib ± rituximab with FCR. The Phase 3 E1912 study enrolled treatment-naive patients ≤70 years of age without high-risk genetic alterations. Three-year results showed that continuous ibrutinib plus rituximab was associated with improved PFS and OS versus FCR. However, the OS benefit is questioned by some experts because of deaths unrelated to treatment or disease and because 31 % of FCR patients did not complete all six treatment cycles. The PFS benefit was more evident in patients with unmutated *IGVH*, with a PFS at 5 years of 75 % in the IR group versus 33 % in the FCR group. The incidence of Grade ≥3 adverse events was similar in both groups, while Grade ≥3 cytopenias and infectious complications were less common with IR than with FCR.

The open-label, multicenter, Phase 3 FLAIR trial,^32^^,^^33^ in two of its four arms, compared 6 years of ibrutinib plus rituximab (IR) versus FCR as first-line treatment in patients with a median age of 62 years without del(17p). IR showed higher 5-year PFS rates compared to FCR, regardless of *IGHV* mutation status. No OS benefit was observed. Median PFS was not achieved in the IR arm and was 67 months in the FCR arm. In addition, at 3 years, 58 % of patients in the ibrutinib-venetoclax arm discontinued therapy due to undetectable MRD. After 5 years of ibrutinib-venetoclax therapy, 66 % of patients had undetectable MRD in BM and 93 % had undetectable MRD in PB.

In general, Grade 3 or greater adverse events were less common in the ibrutinib arms compared to the chemotherapy arms, and adverse events of any grade associated with ibrutinib were consistent across studies. The most common adverse events of any grade were diarrhea, hemorrhage, fatigue, nausea, cough, pyrexia, anemia, rash, thrombocytopenia, and neutropenia. The most common Grade ≥3 events were neutropenia, anemia, pneumonia, thrombocytopenia, hypertension, and diarrhea. Atrial fibrillation occurred in approximately 5–10 % of patients, and 3–8 % of patients developed Grade ≥3 atrial fibrillation. All adverse events should be managed according to institutional therapeutic measures, and multidisciplinary follow-up with a cardio-oncologist is recommended for cardiovascular events.

The introduction of the second-generation BTKis, acalabrutinib and zanubrutinib, provided additional treatment options for CLL in both first line and relapsed/refractory settings, demonstrating greater selectivity for BTK with fewer off-target effects. Follow-up data suggest a lower risk of cardiovascular events compared to ibrutinib.

Acalabrutinib monotherapy was evaluated in relapsed or refractory CLL in the ASCEND trial, which demonstrated its superiority over idelalisib-rituximab or BR, regardless of the presence of *TP53* alterations. The ELEVATE-TN study compared acalabrutinib ± obinutuzumab (Acala±*O*) with chlorambucil + obinutuzumab (CBL+*O*) in previously untreated CLL patients.^49^ Median follow-up was 28.3 months with PFS rates of 93 %, 87 % and 47 % for Acala+*O*, Acala monotherapy and CBL+*O*, respectively. Median PFS was 22.6 months in the CBL+*O* arm and was not achieved in the Acala±*O* arms. There was no statistically significant difference in PFS between the Acala+*O* and Acala monotherapy arms and were beneficial for patients with *TP53* alterations. At the 5-year update, the PFS rate was 71 % for the Acala±*O* arm versus 18 % for CBL+*O* in patients with del(17p) and/or mutated *TP53*.

The ELEVATE-RR trial^50^ compared acalabrutinib with ibrutinib in relapsed or refractory CLL patients with at least one high-risk genetic alteration (mutation and/or del(17p/*TP53*) or del(11q)). At a median follow-up of 40.9 months, acalabrutinib demonstrated non-inferiority to ibrutinib in terms of efficacy and was associated with lower rates of cardiovascular (hypertension and atrial fibrillation) and non-cardiac events (diarrhea, myalgia/arthralgia and bleeding), suggesting that greater BTK selectivity may reduce off-target effects, resulting in an improved clinical safety profile. However, acalabrutinib was associated with higher rates of headache and cough compared to ibrutinib.

The Phase 3 SEQUOIA study evaluated zanubrutinib in previously untreated CLL/SLL patients aged ≥ 65 years who were ineligible for FCR.^51^ The patients were divided into two cohorts: Cohort A - patients without del(17p) randomized to receive zanubrutinib or BR; Cohort B (non-randomized) - patients with del(17) received zanubrutinib monotherapy. In cohort A, with a median follow-up of 26.2 months, the 24-month PFS rate was 85.5 % for zanubrutinib versus 69.5 % for BR. PFS was also superior in the zanubrutinib arm irrespective of *IGHV* status, with an acceptable safety profile. Zanubrutinib was compared to ibrutinib in patients with relapsed or refractory CLL in the Phase 3 ALPINE study.^52^^,^^53^ Zanubrutinib was superior to ibrutinib with 2-year PFS rates of 78.4 % versus 65.9 % (*p*-value = 0.002); OS was not achieved in either treatment arm. The safety profile of zanubrutinib showed fewer serious adverse events and treatment discontinuations compared to ibrutinib. The incidence of Grade ≥3 hypertension was higher with zanubrutinib compared to ibrutinib, but the incidence of any grade atrial fibrillation was lower. Neutropenia occurred in 29 % of patients treated with zanubrutinib. To date, no clinical trial has directly compared acalabrutinib with zanubrutinib. Real-world evaluations of the efficacy and safety of second-generation covalent BTKi are ongoing, with preliminary data currently supporting the results of Phase 3 trials.

A recent matching-adjusted indirect comparison (MAIC) analysis^54^ found that acalabrutinib and zanubrutinib have similar efficacy in relapsed or refractory CLL based on PFS. While adverse event rates were generally comparable, acalabrutinib showed lower rates of serious adverse events, Grade ≥3 hypertension, hemorrhage, and dose reductions. The strength of the study lies in its adherence to MAIC methodology and the minimal impact of matching on acalabrutinib outcomes, reflecting the similarity between ALPINE and ASCEND trials.

### Non-covalent Bruton's tyrosine kinase inhibitors

Covalent BTKis have demonstrated high efficacy and, in many cases, long-term disease control. Because inhibition must be maintained indefinitely to achieve and maintain clinical response, there is a prolonged exposure period during which adverse events and the development of resistance to these agents may occur. Several mechanisms of resistance have been identified, many of which involve mutations in the *BTK* gene or related genes. The mutation at the C481S residue of *BTK* is the most common and occurs at the site where the covalent inhibitor binds to *BTK*, preventing this binding. Another mutation, almost always synchronic with *BTK* mutations, PLCG2, although less common, can activate alternative signaling pathways that bypass the need for BTK. In addition to *BTK* mutations, secondary mutations in other genes related to the B cell receptor pathway or parallel pathways can contribute to the emergence of resistance.

Third-generation non-covalent BTKis have been developed to overcome resistance to covalent BTKis while exhibiting a favorable safety profile. Data show that the mutation at the C481S residue was successfully overcome by non-covalent BTKis, but others such as L528W (frequent after zanubrutinib) and T474I (frequent after acalabrutinib) are not.

Pirtobrutinib showed promising results in a Phase 1/2 study in 276 patients with previously treated CLL/SLL, with an overall response rate of 74 % and a median PFS of 19.4 months. Pirtobrutinib is currently being evaluated in the Phase 3 BRUIN CLL-321 study in populations with prior exposure to BTKis compared to BR or R-idelalisib. A total of 338 patients with a median age of 66 years were equally randomized to two arms, 50 % of patients were also previously treated with venetoclax, and high-risk features were ubiquitous in both arms. At 18 months of follow up, pirtobrutinib demonstrated superior median PFS with 14 months versus 8.7 months as assessed by an Independent Review Committee. No OS survival difference was observed with 73.4 % OS in the pirtobrutinib arm with a 76 % crossover rate from the Standard of care arm likely impacting these data. Although infections were more common in the pirtobrutinib arms, when these data were adjusted for drug exposure time, similar infection rates were documented in the pirtobrutinib arm. Adverse events of interest for pirtobrutinib were consistent with the BTKi class with fewer cases of atrial fibrillation and hypertension compared to cBTKi, but also with a shorter median exposure time.

### Bruton's tyrosine kinase degraders

While currently approved BTKis, such as ibrutinib, work by reversibly or irreversibly binding to BTK to modulate its signaling activity, they do not eliminate the protein itself. Instead, they suppress BTK-mediated survival pathways in malignant B cells, leading to apoptosis.

In contrast, BTK degraders represent an emerging class of therapeutic agents that not only inhibit BTK function, but also actively induce its degradation via the proteasome. These agents use targeted protein degradation (TPD) technology, such as proteolysis-targeting chimeras (PROTACs), to recruit ubiquitin ligases that tag BTK for proteasomal degradation. By eliminating the BTK protein rather than merely inhibiting its activity, BTK degraders may overcome resistance mechanisms associated with BTKis, particularly mutations such as *BTK* C481S that confer resistance to covalent BTKis. However, early data suggest that some non-covalent BTKi resistant mutations, such as A428D, may also confer resistance to BTK degraders, highlighting the need for further investigation into their clinical utility.

Although BTK degraders are not yet approved for clinical use, early phase studies suggest that they may provide deeper and more sustained inhibition of BTK-driven signaling, potentially expanding treatment options for patients with relapsed or refractory CLL and other B-cell malignancies.

With the development of novel BTK-targeting strategies, another critical pathway in B-cell malignancies is the BCL-2-regulated apoptotic machinery, which is effectively targeted by BCL-2is such as venetoclax.

### B-cell lymphoma 2 inhibitors

The B-cell lymphoma 2 (Bcl-2) family of proteins are key regulators of the apoptotic process. The Bcl-2 family includes pro-apoptotic and pro-survival proteins. Shifting the balance toward the latter is an established mechanism by which cancer cells evade apoptosis. Bcl-2, the founding member of this protein family, is encoded by the *BCL2* gene, first described in follicular lymphoma as a result of translocations involving chromosomes 14 and 18, leading to protein overexpression. Venetoclax is a BH3 mimetic that inhibits Bcl-2, promoting apoptosis by releasing pro-apoptotic proteins. It effectively suppresses the growth of Bcl-2-dependent tumors in vivo while sparing human platelets, unlike navitoclax, which was previously tested but did not reach the market due to dose-limiting thrombocytopenia. A single oral dose of venetoclax in three patients with refractory CLL resulted in tumor lysis within 24 h. To mitigate this risk, a stepwise dose escalation regimen was introduced, increasing weekly from 20 mg to 50 mg, 100 mg, 200 mg, and finally to 400 mg over 4–5 weeks. After completing the ramp-up phase, patients continued on 400 mg daily until disease progression or unacceptable toxicity occurred. In the pivotal Phase 1/2 clinical trial, 56 patients received venetoclax in one of eight dose groups ranging from 150 to 1200 mg per day.^55^ In an expansion cohort, an additional 60 patients received venetoclax with progressive weekly dose escalation up to 400 mg per day. Most patients had received multiple prior therapies and 89 % had poor prognostic clinical or genetic features. Venetoclax was effective at all dose levels. Clinical tumor lysis syndrome occurred in three of 56 patients in the dose-escalation arm, with one death. After adjustments to the dose-escalation schedule, none of the 60 patients in the expansion cohort experienced clinical tumor lysis syndrome. No maximum tolerated dose was observed. Of the 116 patients who received venetoclax, 92 (79 %) responded. Response rates ranged from 71 to 79 % in patients with poor prognosis, including those with fludarabine resistance or del(17p) or unmutated *IGHV*. Complete remissions occurred in 20 % of patients, including 5 % of remissions with MRD negativity. The 15-month PFS estimate for the 400 mg dose group was 69 %. Another study was conducted in 107 patients with relapsed or refractory del(17p) CLL. At a median follow-up of 12.1 months, 85 patients (79.4 %) achieved investigator-driven CR. The most common Grade 3–4 adverse events were neutropenia (40 %), infection (20 %), anemia (18 %) and thrombocytopenia (15 %). Serious adverse events occurred in 55 % of patients, with the most common (≥5 % of patients) being fever and autoimmune hemolytic anemia (7 % each), pneumonia (6 %), and febrile neutropenia (5 %). Eleven patients in the study died within 30 days of the last dose of venetoclax, seven due to disease progression and four due to adverse events (none considered treatment-related). Together, the results of the two studies demonstrate that venetoclax monotherapy is active and well tolerated in patients with relapsed or refractory del(17p) CLL, providing a new therapeutic option for this population with a very poor prognosis.

The Phase 3 CLL14 trial, a multicenter, randomized, open-label study conducted at 196 research centers in 21 countries,^56^ enrolled treatment-naïve CLL patients over 65 years of age and/or with comorbidities (CIRS score greater than 6). Patients were randomized to receive venetoclax (orally initiated on Day 22 of Cycle 1 [28-day cycles] with a 5-week escalation [20 mg, 50 mg, 100 mg, 200 mg, then 400 mg daily for 1 week], continuing at 400 mg daily until completion of cycle 12; combined with intravenous obinutuzumab for six cycles starting with 100 mg on Day 1 and 900 mg on Day 2 [or 1000 mg on Day 1], 1000 mg on Days 8 and 15 of Cycle 1, and then 1000 mg on Day 1 of Cycles 2 through 6) or chlorambucil with obinutuzumab (oral chlorambucil at 0. 5 mg/kg body weight on Days 1 and 15 of each cycle for 12 cycles in combination with the same obinutuzumab regimen). A total of 432 patients were randomized (venetoclax and obinutuzumab: *n* = 216; chlorambucil and obinutuzumab: *n* = 216). At a median follow-up of 76.4 months,^57^ PFS remained superior in the venetoclax-obinutuzumab arm compared to the chlorambucil-obinutuzumab arm (median, 76.2 versus 36.4 months, *p*-value <0.0001). Similarly, time to next treatment (TTNT) was significantly longer (6-year TTNT: 65.2 % versus 37.1 %, respectively; *p*-value <0.0001). The most common Grade 3 or 4 adverse event in both groups was neutropenia (53 % in the venetoclax-obinutuzumab arm and 48 % in the chlorambucil-obinutuzumab arm). In an update of the study 2 years after completion of treatment, patients who received venetoclax-obinutuzumab continued to show a significant PFS benefit with no new evidence of associated adverse events, making it an excellent finite therapy option for this elderly and/or comorbid patient population.

In the Phase 3 GAIA (CLL13) clinical trial,^58^ patients with CLL who were fit and had no *TP53* gene abnormalities were randomized in a 1:1:1 ratio to receive six cycles of chemotherapy: one to six cycles of chemoimmunotherapy (fludarabine-cyclophosphamide-rituximab or bendamustine-rituximab) or 12 cycles of venetoclax-rituximab, venetoclax-obinutuzumab or venetoclax-obinutuzumab-ibrutinib. Of the 926 patients randomized, 229 received chemoimmunotherapy, 237 received venetoclax-rituximab, 229 received venetoclax-obinutuzumab and 231 received venetoclax-obinutuzumab-ibrutinib. At Month 15, the proportion of patients with undetectable MRD was significantly higher in the venetoclax-obinutuzumab (86.5 %) and venetoclax-obinutuzumab-ibrutinib (92.2 %) arms compared to the chemoimmunotherapy (52 %) and venetoclax-rituximab (57 %) arms. The 3-year PFS was higher in the venetoclax-obinutuzumab (87.7 %) and venetoclax-obinutuzumab-ibrutinib (90.5 %) arms compared to the chemoimmunotherapy (75.5 %) and venetoclax-rituximab (80.8 %) arms. Grade 3 and 4 infections were more common with chemoimmunotherapy (18.5 %) and venetoclax-obinutuzumab-ibrutinib (21.2 %) than with venetoclax-rituximab (10.5 %) or venetoclax-obinutuzumab (13.2 %). The study suggests that, as in elderly patients, the combination of venetoclax and obinutuzumab appears to be the most effective finite therapy option, offering better response rates, survival, and safety. However, longer follow-up is needed to confirm these results.

The Phase 3 AMPLIFY trial (NCT03836261) evaluated acalabrutinib-venetoclax (AV) and acalabrutinib-venetoclax-obinutuzumab (AVO) versus chemoimmunotherapy (FCR or BR) in treatment-naive CLL patients without *TP53* aberrations.^53^ At a median follow-up of 41 months, both AV and AVO significantly improved PFS compared to chemoimmunotherapy, with median PFS not reached in either acalabrutinib-containing arm. The 36-month PFS rates were 76.5 % (AV), 83.1 % (AVO) and 66.5 % (FCR/BR). Overall response rates were also higher with AV (92.8 %) and AVO (92.7 %) versus FCR/BR (75.2 %). Grade ≥3 neutropenia was the most common adverse event, and serious adverse events were most common in AVO-treated patients (38.4 %). These findings support acalabrutinib-based regimens as effective, chemotherapy-free alternatives in treatment-naive CLL, with AV offering a favorable safety/efficacy balance and AVO achieving the highest PFS but with more toxicity.

### Comorbidities and patient preference

Selection of the optimal therapy for CLL continues to be guided by a personalized approach that considers both disease biology and patient-specific factors. Comorbidities play a critical role in treatment selection, as many patients with CLL are elderly and have cardiovascular, renal, or autoimmune diseases that may limit the use of certain therapies. For example, patients with a history of atrial fibrillation or bleeding disorders may not tolerate BTKis, particularly ibrutinib, while patients with renal impairment may require dose adjustments or alternative regimens to BCL-2is. In addition, patient preferences have a significant impact on treatment decisions, as considerations such as route of administration (oral versus intravenous), treatment duration (fixed duration versus continuous therapy) and side effect profiles affect adherence and quality of life. The increasing use of MRD-driven strategies also allows for more individualized treatment durations, allowing for discontinuation of therapy in patients who achieve deep remissions while maintaining durable disease control.^59^

### Infectious complications in CLL

Given the profound immune dysregulation associated with CLL and the immunosuppressive effects of targeted therapies, infection prevention remains a cornerstone of patient management. Vaccination strategies have gained prominence, with strong recommendations for the administration of inactivated vaccines, including influenza, pneumococcal, and COVID-19 vaccines, to all patients with CLL, ideally prior to treatment initiation. While response rates to vaccines may be suboptimal due to underlying immune dysfunction, newer strategies such as booster doses and passive immunization with monoclonal antibodies against SARS-CoV-2 have shown promise in improving protection. In addition to vaccination, prophylactic antimicrobials, including antiviral agents (e.g., acyclovir for herpesvirus reactivation) and *Pneumocystis jirovecii* pneumonia (PJP) prophylaxis in selected patients receiving B-cell depleting therapies, remain essential to reduce infectious complications. Regular immunoglobulin replacement therapy is being considered for patients with recurrent infections and hypogammaglobulinemia, further highlighting the importance of a proactive, individualized approach to infection management in CLL.

### Treatment in public or private centers in Brazil and other less-resourced countries

Due to systemic inequities, hematologists in Brazil face additional challenges in determining the best treatment regimen for CLL patients. Access to novel therapies is highly inequitable between public and private healthcare institutions, with significant implications for the efficacy and tolerability of CLL treatments.^60^

In the first published analysis of the Brazilian Group of CLL,^61^ the median follow-up time of 1903 patients was 36 months (range: 3–155 months). Treatment-free survival at 3 and 5 years was 44 % and 32 %, respectively with advanced Binet staging showing a strong correlation with inferior survival.

Patients from public and private institutions were compared in an analysis of the Brazilian CLL Registry.^62^ Of 3326 patients, 81 % were in public hospitals and 19 % in private hospitals. Public hospital patients were older (median age 66 years versus 63 years in private hospitals), had more advanced disease (44 % versus 33 % Binet B or C), and more frequently had elevated creatinine levels (18 % versus 10 %). Prognostic markers were evaluated more frequently in private hospitals: FISH for del(17p) (45 % versus 10 %), *IGHV* mutation (19 % versus 6 %), karyotype (24 % versus 12.5 %), and beta-2 microglobulin (47 % versus 32 %). The frequency of FISH-positive del(17p) was similar (10.5 % versus 9 %), as was the frequency of unmutated *IGHV* (50 % versus 56 %). Due to missing data, only 432 patients (13 %) were stratified by CLL-IPI: 175 (40 %) with low/intermediate scores and 257 (60 %) with high/very high scores.^62^ High-risk CLL-IPI patients were more likely to be found in public hospitals (69 % versus 45 %).

Regarding treatment, chlorambucil or fludarabine was the most commonly used first-line therapy (chlorambucil: 41 %; fludarabine: 38 %). Anti-CD20 monoclonal antibodies were used in only 36 % of cases (rituximab: 32 %; obinutuzumab: 4 %). New agents were used in only 5 % of cases. Public hospitals were less likely to use fludarabine (36 % versus 48 %) and anti-CD20 monoclonal antibodies (26 % versus 75 %). Surprisingly, the majority of patients with del(17p) or *TP53* mutations (69 %) received chemoimmunotherapy as first-line therapy. Median follow-up was 39 months, and overall survival was 71 % at 5 years, which was worse in public hospitals (68 % versus 82 %). These data show significant differences between patients treated in public and private hospitals likely due to a more advanced initial presentation and lack of access to appropriate testing and therapies.^62^

**Figure 1:**
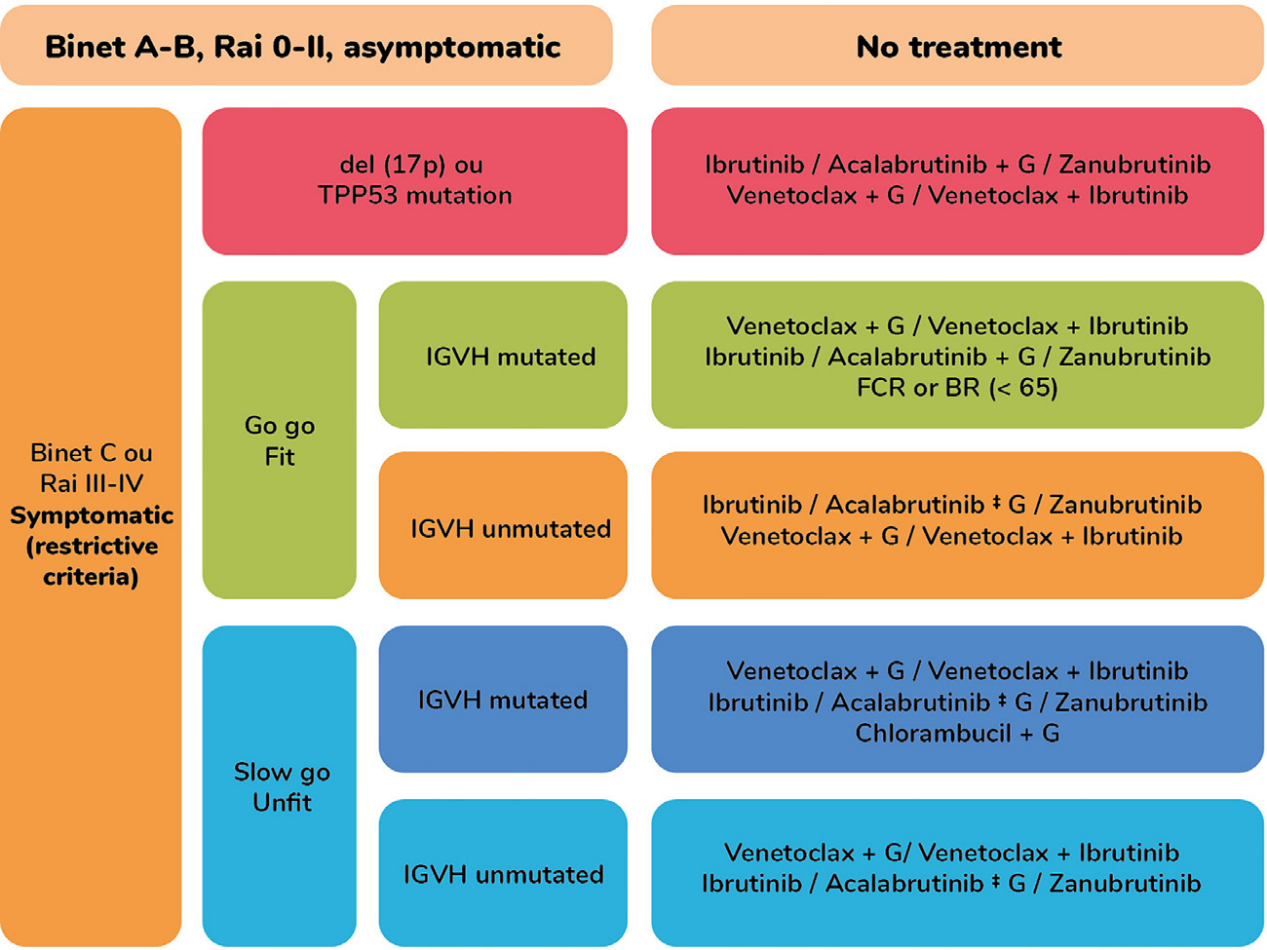
Brazilian Group of CLL established treatment recommendations.

In 2025, the Brazilian Group of CLL established treatment recommendations based on the accumulated evidence to date, which are presented in Figure 1.

## Conclusions

CLL remains the most common form of leukemia in adults and presents complex clinical challenges due to its heterogeneous nature and variable response to treatment. Research highlights several key points:

1. Epidemiology and diagnosis: CLL typically affects older adults, with a significant proportion of patients asymptomatic at diagnosis. Identification of monoclonal B lymphocytes with specific immunophenotypic markers is critical for diagnosis, highlighting the importance of advanced diagnostic techniques in differentiating CLL from other B-cell malignancies.
2. Prognostic factors: Prognostic stratification remains critical, with *IGHV* mutation status and chromosomal abnormalities, mainly deletions involving *TP53*, serving as significant indicators of disease aggressiveness and treatment resistance. The integration of clinical staging systems with genetic profiling is essential to tailor treatment strategies.
3. Treatment advances: The introduction of targeted therapies, such as BTK inhibitors and BCL-2 inhibitors, has revolutionized the treatment of CLL. These therapies show superior efficacy compared to traditional chemotherapy, especially in high-risk populations. However, the need for careful patient selection and consideration of comorbidities is paramount to optimize outcomes and minimize treatment-related adverse effects.
4. Healthcare disparities: Analysis of access to care in Brazil highlights the disparities between public and private healthcare systems, revealing significant differences in patient demographics, treatment modalities and prognostic assessments. These disparities call for strategic interventions to improve access to effective therapies, especially for vulnerable populations.
5. Future directions: Ongoing research into measurable residual disease (MRD) assessment and novel therapeutic agents holds promise for further improving outcomes in CLL. Further development of treatment protocols and incorporation of MRD assessment into clinical practice may improve long-term survival and quality of life for patients.

In conclusion, a comprehensive understanding of the biological basis of CLL, coupled with advances in diagnostic and therapeutic approaches, is critical to improving patient care. Future efforts should focus on bridging gaps in care and optimizing treatment protocols to ensure equitable access to effective therapies for all CLL patients.

## Conflicts of interest

The authors declare no conflicts of interest.
